# Preclinical research models for evaluating the biocompatibility of bioresorbable metallic cardiovascular stents: A comparative review

**DOI:** 10.1016/j.bioactmat.2025.09.004

**Published:** 2025-09-14

**Authors:** Samuel Hansen, Thuy Anh Bui, Xiaoxue Xu, Kristine McGrath

**Affiliations:** a School of Life Sciences, Faculty of Science, University of Technology Sydney, 745 Harris St, Ultimo, NSW, Australia, 2007; b School of Biomedical Engineering, Faculty of Engineering and Information Technology, University of Technology Sydney, 81-113 Broadway, Ultimo, NSW, Australia, 2007; c Ingham Institute for Applied Medical Research, 1 Campbell St, Liverpool, NSW, Australia, 2170; d Faculty of Medicine and Health, University of New South Wales, Cnr High St & Botany St, Kensington, NSW, Australia, 2033

**Keywords:** Bioresorbable stents, Atherosclerosis, Preclinical models, Biodegradable metals

## Abstract

Cardiovascular stents are widely used to treat atherosclerosis by relieving vascular obstruction and providing structural support after coronary angioplasty. Bioresorbable metallic stents represent a promising alternative to conventional corrosion-resistant stents, which are linked to late-stage complications such as in-stent restenosis and thrombosis. Due to the diversity of stent materials and designs, rigorous evaluation of their interactions with the vascular environment in relevant preclinical models is essential before clinical translation. However, current studies employ highly variable in vitro cell systems, in vivo animal models, and experimental assays to assess biocompatibility, making it difficult to draw definitive conclusions about candidate designs. This review outlines the current landscape of bioresorbable metallic stents, critically examines the strengths and limitations of preclinical models described in the literature and in international guidelines, and provides recommendations to guide future research in this rapidly evolving field.

## Highlights

• Review highlights inconsistent preclinical models for stent evaluation.
• Assays used to assess the biocompatibility of bioresorbable metals were discussed.
• Current in vitro cellular models can inaccurately replicate the human vasculature.
• In vivo animal models are promising, however have a high economic and ethical cost.
• Future prospect of in vitro 3D cell culture models are discussed.

## Graphical abstract

**Figure:**
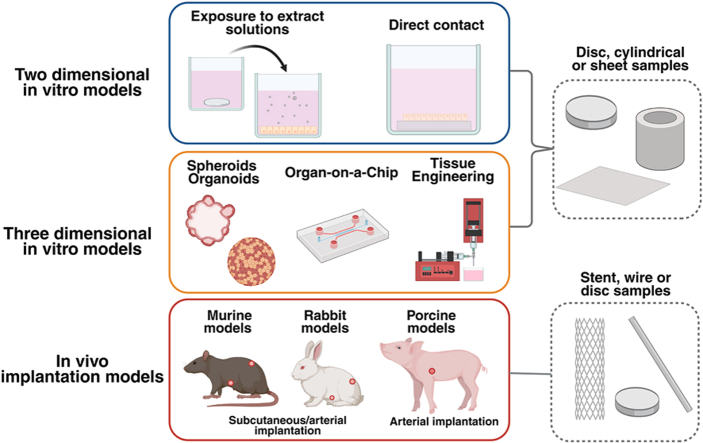

## 1 Introduction

**Fig. 1:**
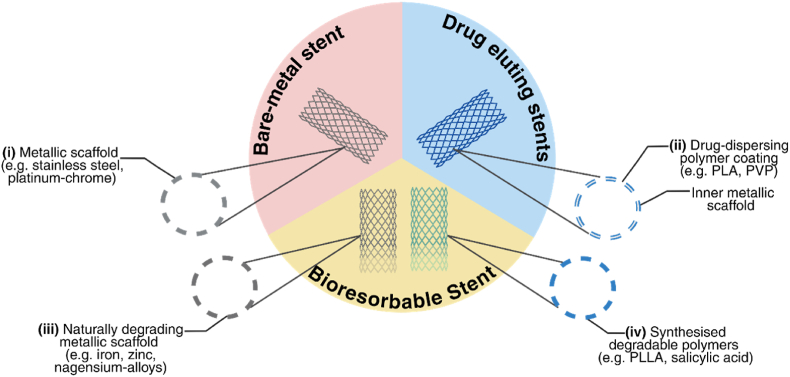
Three main categories of cardiovascular stents. (i) Bare-metal stents, comprised of a metallic scaffold of corrosion resistant metals (such as stainless steel) or metallic alloys (such as platinum/cobalt-chrome alloys) were the first model of stent developed for use. These stents were associated with a high rate of complications post-implantation including the restenosis of vessels. To address this, (ii) drug-eluting stents, comprised of a metallic scaffold coated with a drug-eluting polymer (such as poly-lactic acid (PLA), polyvinylpyrrolidone (PVP), poly(vinylidencefluoride-cohexafluoropropenen) (PDF-HFP). Bioresorbable stents further provide an alternative to DES and BMS, comprised of either (iii) naturally degrading metals (such as magnesium, zinc or iron-based alloys), or (iv) polymers (such as poly-L-lactic acid or salicylic acid) due to their capability to degrade following implantation.

Despite significant advancements in the diagnosis and management of cardiovascular diseases, coronary artery disease (CAD) remains the leading cause of death worldwide, accounting for more than 9 million deaths each year [1]. To reduce the burden of CAD, a range of therapeutics have been developed, including medications such as statins or beta blockers, as well as surgical interventions such as percutaneous coronary interventions and stent implantation [2,3]. Among these, cardiovascular stents, introduced in the late 1980s, have since become one of the most commonly used treatment options to reduce the risk of acute cardiovascular events in advanced CAD cases where medicinal therapy alone is insufficient [4,5]. To date, several types of stents with distinct properties have been developed, including bare-metal stents (BMS), drug-eluting stents (DES), and bioresorbable stents (BRS) (Fig. 1.), [4].

Bare-metal stents, the first type of cardiovascular stents developed, are manufactured solely from corrosion-resistant metals such as stainless steel or cobalt-based alloys [[5], [6], [7]]. Initially, these stents demonstrated strong mechanical properties and proved effective in treating CAD [[8], [9], [10], [11]]. However, the implantation of BMS typically results in post-operative complications such as blood vessel restenosis and thrombus formation [[8], [9], [10], [11]]. To address these issues, DES, polymer or metal-based stents coated with anti-inflammatory drugs were introduced with the aim of decreasing neointimal proliferation and short-term restenosis [9,11,12]. Whilst their short-term performance was promising, there is a high likelihood for DES to induce late-stage inflammatory responses and neo-atherosclerosis [[13], [14], [15]]. Recent years have seen growing interest in the use of BRS, a newly emerging type of stent, which can gradually degrade within the arteries over time [4,16]. This degradable feature offers several advantages, such as addressing atherosclerotic plaque growth, preventing vessel collapse after percutaneous intervention, promoting arterial repair and eliminating the need for surgical removal if restenosis occurs [16,17].

Whilst BRS offer significant advantages over BMS and DES, extensive evaluation of their properties and clinical outcomes is required [18]. Potential BRS must be designed to provide mechanical support to the arterial wall during remodelling, degrade at a consistent rate over 12–24 months post-implantation and remain compatible within the cardiovascular environment. To achieve these outcomes, specific design parameters for an ideal BRS have been proposed. These include optimal mechanical properties, such as a high tensile strength (>300 MPa), elasticity (elongation to failure >15–18 %) and a degradation rate ranging from 0.02 to 0.04 mm/year [16,19]. Whilst it is pertinent to characterise the dynamic degradation of metallic BRS, as well as the subsequent changes to the mechanical properties of the stent, another paramount characteristic of BRS is that they must remain biocompatible once implanted [16,19]. Unlike the mechanical properties or degradation behaviour of BRS which have been quantified with specific values such as tensile strength or elasticity, or by evaluating the dynamic ion-release from candidate metals, evaluating the biocompatibility of BRS requires both quantitative and qualitative assessments due to the complex interactions and mechanisms occurring within biological tissues which cannot be fully described by numerical values alone [16,19].

The definition of biocompatibility has undergone several revisions, with the currently agreed definition of biocompatibility by Williams [20] in the 1970s as, “… the ability of a material to perform with an appropriate host response in a specific situation”. To contextualise this definition within the field of biomaterials, international and nationally equivalent standards have been established to provide a research framework when evaluating the biocompatibility of candidate BRS [[21], [22], [23], [24], [25], [26]]. Combined, the current accepted understanding is that for a BRS to be biocompatible, it is essential that the implanted BRS does not provoke any adverse immune responses or induce systemic toxic effects within the arterial environment to maintain vascularity and prevent restenosis [16,21].

Various materials have been explored to meet the criteria mentioned above, ranging from synthetic polymers to metallic alloys [17,19,27]. Initially, synthetic polymers such as poly-l-lactic acid (PLLA) and poly (lactic-co-glycolic acid) (PLGA) were favoured for BRS development, but their mechanical strength has proven to be insufficient, resulting in premature stent fracture [27,28]. In contrast, BRS made from metallic alloys have been demonstrated to possess superior mechanical properties, with reported tensile strengths 3–4 times higher than BRS produced from polymers, whilst remaining to degrade within the ideal 12-24-month time period [17,19,27]. Using metals to produce BRS is thus advantageous due to their ability to provide mechanical support which is comparable to traditional BMS, whilst safely degrading within the body at an acceptable rate [16].

### 1.1 Improvements to the design and structure of metallic BRS

Early research into metallic BRS primarily has emphasised the use of pure metals, particularly iron (Fe), magnesium (Mg) and zinc (Zn), which are present within the body as essential nutrients for human health and play crucial roles in various physiological functions, which support their compatibility within the arterial environment [19,[29], [30], [31], [32]]. Of these metals, BRS comprised of magnesium and iron have been most extensively researched, due to their ideal mechanical properties and low potential to induce minimal host responses within the body once implanted [27,28]. Iron-based BRS have been found to possess high mechanical strengths, with ultimate tensile strengths (UTS) of 230–245 MPa [33]. However, pure-iron BRS were found to incompletely and inconsistently degrade once implanted, resulting in an excess of degradation products released around the site of implantation, which raised concerns about their long-term use and safety [34,35]. Magnesium-based BRS have also been extensively investigated due to magnesium's well-characterised roles within the body, including its involvement in cell signalling pathways and cellular metabolism, making it a highly biocompatible material [36,37]. These magnesium BRS were, however found to have an unsatisfactory, high degradation rate (0.3–0.6 mm/year) [16,38]. This resulted in the stent's mechanical integrity becoming compromised, as well as the release of hydrogen gas into the bloodstream, potentially affecting patient safety [16,38]. More recently, zinc has emerged as a promising bioresorbable material for cardiovascular applications due to its ideal corrosion behaviour (0.015–0.2 mm/year) and biocompatibility comparable to that of magnesium-based BRS [19,39]. Despite this, stents made from pure zinc have poor mechanical strength (UTS 90–200 MPa), limiting its potential as a candidate BRS material [19,40]. Therefore, although metallic BRS of pure metals offer distinct advantages, they also present significant complications that hinder their overall suitability as BRS. To address the limitations of single-metal BRS, research has led to the development of metallic alloys by combining pure metals with additional metallic elements such as copper (Cu), lithium (Li), silver (Ag), magnesium (Mn) or aluminium (Al) [37,[41], [42], [43]]. Additionally, there has been a growing incidence of the use of coatings to modify the surface of metallic BRS [43]. These include ion injection/deposition coatings upon which metallic ions are sputtered on the surface of the alloy, or chemical conversion in which phosphates and carbonates are chemically deposited onto alloys to modify the degradation of the stent [[43], [44], [45]]. Drug-releasing coatings have further begun to be utilised in clinical trials to reduce post-surgical host responses to the implanted candidate BRS and in turn, improve its clinical performance [43,46].

These modified metals have demonstrated both superior mechanical properties and degradation rates to pure-metal BRS, which enhances their therapeutic potential and success once implanted [16,32,47]. Research into metallic alloys has been promising, with several metallic BRS having advanced to clinical trials [[48], [49], [50]]. Whilst these trials demonstrate that BRS have adequate clinical performance and safety comparable to that of a DES, they have also been shown to induce adverse complications such as scaffold-induced thrombosis or late-stage neointimal hyperplasia [49,51]. Thus, whilst research into metallic BRS has been extensive, their design continues to be optimised and refined to ensure safer and enhanced clinical outcomes [52,53].

### 1.2 Assessing the biocompatibility of bioresorbable metallic alloys

Alongside evaluating the mechanical properties and degradation of candidate metallic BRS, it is crucial that, before clinical use, all bioresorbable metals undergo a multitude of rigorous tests to evaluate their biocompatibility to ensure that any adverse effects following implantation are identified in advance [42]. Guidelines such as the International Organisation for Standardisation (ISO) series 10993 (or nationally equivalent standards) have been created to provide suggestions for the experimental design of studies that intend to evaluate the biocompatibility of biomedical materials including orthopaedic, dermatological and cardiovascular implants [21]. Specifically, ISO series 10993 details key biological responses that should be tested to comprehensively characterise how the potential biomedical devices interact with bodily tissues. As detailed in Table 1, these responses include evaluating cellular and systemic toxicity, immunogenic effects induced by materials following exposure to tissue, haemocompatibility (the compatibility of materials with the blood and its components), as well as the genotoxic and carcinogenic potential of materials [[21], [22], [23], [24], [25], [26],^54^]. In addition to addressing the key aspects of biocompatibility, ISO series 10993 also provides recommendations on the appropriate use of preclinical experimental models for evaluating biocompatibility, including both in vitro and in vivo approaches [21,24,26].

**Table 1:** Key categories of biocompatibility to evaluate pertaining to metallic BRS development.

Aspect of Biocompatibility	Conditions of Cell Cultures	Relevant International Standard (ISO)	Refs
Toxicity	The extent to which materials induce cytotoxic, systemic or chronic toxic effects	10993–5	[25,26]
		10993–11	
Immunogenicity	The potential of a material to induce immune responses and irritation	10993–6	[24,54]
		10993–20	
Haemocompatibility	The effects of materials on the blood and its components	10993–4	[23]
Genotoxicity	The potential of materials to induce genetic mutations or chromosomal damage	10993–3	[22]
Carcinogenicity	The tumorigenic potential of materials following implantation	10993–3	[22]

These experimental guidelines serve as an effective tool for researchers by providing experimental frameworks for evaluating the biocompatibility of metallic BRS both in vitro and in vivo. As such, these guidelines are regularly referred to in previous literature that evaluated the biocompatibility of iron, magnesium, zinc and other metallic BRS. Table 2 provides an overview of the current reported biocompatibility of metallic BRS, including the preclinical models used to reach these conclusions, as reviewed in detail by Chen et al. [53] and Oliver et al. [46]. Despite the availability of these guidelines, their broad categorisation of biomedical implants means the recommendations remain generalised and non-specific. Consequently, the choice of the preclinical models in previous studies has left to the discretion of the investigator, based on their expertise and knowledge of the candidate biomaterial [21,24,26]. This has led to considerable variability in the experimental assays, cell lines and animal models used in studies reporting on the biocompatibility of BRS, creating challenges in comparing findings across the literature [55,56]. This review thus aims to summarise, compare and evaluate the suitability of various preclinical research models currently reported for evaluating the biocompatibility of candidate bioresorbable metallic stents. By consolidating this information, it can help guide the development of more coherent and standardised experimental protocols for evaluating the biocompatibility of future candidate metals.

**Table 2:** Reported biocompatibility of previous metallic BRS.

Metal	Experimental Models Utilised	Pre-clinical observations	Clinical outcomes	Refs
Magnesium, Mg-based alloys	In vitro, in vivo (mice, rats, pigs, rabbits) & clinical studies.	Low cytotoxicity, cell attachment or recruitment; minimal inflammatory responses, neointimal activation or morphological changes; no significant haemolysis or thrombogenesis.	Good procedural success rate; acceptable safety and performance. Higher degradation, occasional excessive release of breakdown products and hydrogen gas. Design of optimal stent remains ongoing.	[36,46,53,57]
Iron, Fe-based alloys	In vitro & in vivo (mice, rats, rabbits, pigs)	Acceptable cytotoxicity, minimal cell attachment of recruitment; minimal inflammatory responses, neointimal activation or morphological changes; no significant haemolysis or thrombogenesis. Slower degradation rate and occasional release of toxic breakdown products.	No current clinical trials	[46,53,58,59]
Zinc, Zn-based alloys	In vitro & in vivo (mice, rats, rabbits, pigs)	Low cytotoxicity, cell attachment or immune cell recruitment; minimal inflammatory responses, neointimal activation or morphological changes; no significant degree of haemolysis or thrombogenesis. Adequate degradation rate, concerns related to mechanical stability and strength whilst degrading.	No current clinical trials	[46,53,[60], [61], [62]]
Molybdenum	In vitro & in vivo (mice)	Low cytotoxicity; minimal inflammatory responses, neointimal activation or morphological changes; no significant degree of thrombogenesis or haemolysis. Adequate degradation rate, behaviour & mechanical strength.	N/A	[46,53,63,64]

## 2 In vitro models to evaluate the biocompatibility of metallic bioresorbable stents

The use of in vitro models for assessing candidate bioresorbable metals is an essential first step in building foundational understanding of the material and evaluating their potential for development into a BRS and eventual clinical implementation [56]. These cellular models serve as a cost-effective and time-efficient method for assessing how candidate metals interact with the surrounding tissue at the site of implantation, as well as the effects of their degradation products [56]. Furthermore, the use of in vitro models allows the identification of any potential complications, such as significant levels of toxicity, inflammation or host responses, to be observed without inducing any unnecessary harm to animal subjects or patients in clinical trials [56]. As a result, only the candidate metals with the highest biocompatibility are advanced to later stages of preclinical research.

**Fig. 2:**
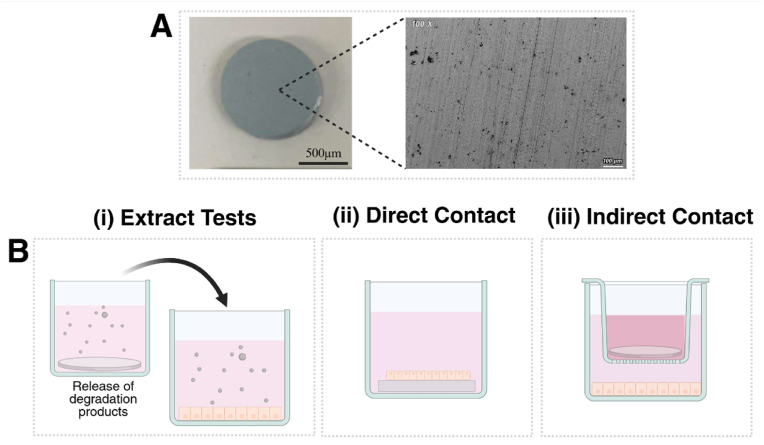
In vitro models to evaluate the biocompatibility of metallic BRS. (A) Macroscopic (left) and high magnification image (right) of sample candidate metals are typically utilised in vitro. High (100X) magnification image obtained with scanning electron microscopy (conducted on a Zeiss EVO electron microscope) from unpublished data from our research team. (B) In vitro experimental models for evaluating biocompatibility consist primarily of (i) extract tests, where cells are cultured in media containing degradation products extracted from candidate metals; (ii) direct contact tests, where cells are cultured directly on the surface of candidate metals; or (iii) indirect contact test, where candidate metals are placed in inserts and positioned above cells to avoid direct contact, allowing degradation products to diffuse through the shared culture media and interact with the cells. S.

For ease of handling in vitro, candidate metals are typically prepared into smaller, flat, cylindrical samples (approximately 5–10 mm in diameter, 2–5 mm in thickness**) (**Fig. 2A**),** rather than using a whole stent [26,45,65,66]. If relevant, any coatings of interest are applied directly onto samples after preparation to ensure their presence during in vitro experiments. To evaluate the various aspects of biocompatibility outlined in Table 1, three types of in vitro models are commonly used to simulate physiological conditions and the release of degradation products from candidate metals: extract tests, direct contact tests and indirect contact tests (Fig. 2B) [26].

Of these aspects of biocompatibility, the toxicity of candidate bioresorbable metals is predominantly evaluated, with the other aspects of biocompatibility being investigated sparsely in vitro [44,45,59,62,[65], [66], [67], [68], [69], [70], [71], [72], [73], [74], [75], [76]]. Additionally, insights gained from cytotoxicity tests can provide indications of further experimental analysis, such as indicating the need to alter the design of the candidate metal or specific aspects of biocompatibility to explore further in depth [44,45,59,62,[65], [66], [67], [68], [69], [70], [71], [72], [73], [74], [75], [76]].

When referring to international guidelines, ISO 10993-5 provides guidelines for evaluating the cytotoxicity of materials using both qualitative and quantitative methods [26]. For a detailed quantitative evaluation of cytotoxicity, ISO 10993-5 states that measurements such as cell death, inhibition of growth, cell-proliferation, protein production, or the metabolic reduction of cellular dyes can further be utilised to evaluate cytotoxicity [26]. As such, cytotoxicity is commonly evaluated using colourimetric tetrazolium-based assays, such as the MTT, XTT, MTS or CCK-8 assays, which rely on a cells’ ability to metabolise dyes into coloured formazan crystals. This process indicates mitochondrial activity, from which cell viability is inferred [44,45,59,62,[65], [66], [67], [68], [69], [70], [71], [72], [73], [74], [75], [76]]. Alternatively, live/dead cellular stains which bind to intracellular and extracellular amines, when paired with fluorescent microscopy or flow cytometric analysis can be utilised to quantify cell death, membrane integrity and determine the stage of the cell cycles at which cells die following exposure to candidate metals to further investigate their cytotoxicity [66]. Cell apoptosis assays have also previously been performed to measure the number of cells that have undergone programmed cell death following exposure to extracts [[61], [62], [63],^67^].

For a qualitative evaluation of the cytotoxicity of candidate metals, cytochemical staining and microscopy can be used to observe general changes in cellular morphology, cellular detachment or lysis following exposure to candidate metals [37,44,45,65,67,74]. These qualitative observations have been assigned a quantitative grading scheme, as detailed in ISO 10993-5, here-in summarised in Table 3, which can be utilised to give a numerical value to the qualitative observations made using both extract and direct contact tests [26].

**Table 3:** Grading system for quantifying cytotoxicity based on qualitative morphological analysis (ISO 10993-5).

Conditions of Cell Cultures	Grade
Little to no granular formation within the cytoplasm; no cell lysis or noticeable reduction of cell growth.	0
>20 % of cells are observed to have become rounded, detached or changed morphologically; little to no granular formation within the cytoplasm; occasional cell lysis or slight inhibition of growth observed	1
>50 % of cells are observed to have become rounded, detached or changed morphologically; no extensive granular formation within the cytoplasm; no extensive cell lysis and >50 % of growth inhibition observed	2
>70 % of cells observed to have become rounded, detached or lysed; cellular layer not destroyed, but <50 % growth inhibition observed.	3
Extensive cellular lysis and almost complete degradation of cellular layers.	4

### 2.1 Evaluating aspects of biocompatibility utilising extract tests

Extract tests are designed to simulate the gradual release of degradation products, primarily metallic ions, that occur as metallic BRS break down following implantation in the vasculature [55]. In these tests, cells are exposed to ‘extracts’: solutions generated by incubating candidate metals in physiological media, which contain breakdown products released from the metal samples whilst degrading (Fig. 2B–i) [77,78]. This setup, therefore, allows observation of cellular responses to the degradation products released by BRS in a controlled setting [26].

In relation to BRS, extracts are prepared by steeping samples of candidate metals in a physiological medium that includes cell culture media, blood or simulated body fluid, at a specific surface area-to-volume ratio of 1.25 cm^2^/mL for 24–72 h (as detailed within ISO 10993-5), under conditions which simulate the clinical conditions of the body (i.e. 37 °C, 5 % CO_2_ atmosphere) [72,73,77,78]. Once prepared, cells are cultured in these extracts at varying concentrations (typically ranging from 5 to 100 % pure extract) for 1–14 days [44,59,65,66,[68], [69], [70], [71], [72], [73], [74], [75],^77^,^78^]. This approach helps determine the optimal concentration of breakdown products that cells can tolerate before significant adverse effects or responses occur [26,72,73,77,78].

In excess, degradation products released from candidate bioresorbable metals can result in elevated intracellular metallic ion levels, disrupting cell signalling and potentially causing programmed cell death or cellular dysfunction [79]. As such, the cytotoxic effects of the degradation products released from candidate bioresorbable metals are typically examined to evaluate if the metal degradation has the potential to induce intracellular metallic ion overload and excessive cell death [44,45,58,59,62,[65], [66], [67], [68], [69], [70], [71], [72], [73], [74], [75]]. This rationale further provides explanation for the dilution of extracts to various concentrations, allowing investigators to determine the threshold at which degradation products begin to induce excessive cell death. These findings can then be used to optimise the design of the metallic BRS for safer and more effective clinical use [44,45,58,59,62,[65], [66], [67], [68], [69], [70], [71], [72], [73], [74], [75]]. Accordingly, extract tests are primarily employed to evaluate the cytotoxicity of candidate metallic BRS (Table 4).

**Table 4:** Common cytotoxicity test methods for evaluating bioresorbable metal using extract tests.

Category of biocompatibility	ISO standard	Biological Response	Relevant tests	Refs
Cytotoxicity	ISO 10993-5	Viability	Metabolism-based assays (MTT, CCK-8, WST-1, WST-8, lactate-dehydrogenase release assays) Cell-proliferation assays (BrdU, WST-8) Live-dead microscopy	[44,45,58,59,62,[65], [66], [67], [68], [69], [70], [71], [72], [73], [74], [75]]
		Morphological changes	Fluorescence microscopy	[37,44,45,65,67,74]
		Programmed cell death	Apoptosis-detection assays Flow Cytometry	[66,74]
		Cell Function	Cell migration, adhesion and spreading tests assays	[80]

Alongside cytotoxicity and cell functionality assays, extract tests have further been utilised to evaluate the immunogenicity of candidate metallic BRS by examining the expression of inflammatory genes (such as IL-8, CCL2, ICAM or VCAM-1) following exposure to extracts, using quantitative real-time PCR [63,67]. Though overall, these aspects of biocompatibility are sparsely investigated in vitro, with investigators preferring to utilise direct contact in vitro models or vivo studies [44,45,70,74,[81], [82], [83], [84]].

Extract tests thus provide valuable insight into cellular responses to breakdown products released from candidate BRS metals when implanted in artificial fluids that mimic cardiovascular environments. However, the experimental design of this model has significant limitations that raise concerns about their reliability – the most notable being the reported discrepancy between the concentration of breakdown products in artificially prepared extracts and their actual release into the bloodstream in vivo [58,85]. Within blood vessels, the continuous flow of blood clears breakdown products from the site where BRS are implanted and degrade, resulting in arterial cells being exposed to constantly changing concentration of these products in vivo [85]. Consequently, extract tests do not perfectly reflect the arterial environment, and findings from these tests should be interpreted with consideration of their limitations and may not, on their own, provide a comprehensive assessment of the biocompatibility of candidate metals.

### 2.2 Evaluating aspects of biocompatibility utilising direct contact tests

For direct contact tests, cells are cultured directly on the surface of samples of the candidate metal itself (Fig. 2B–ii). This provides valuable insight into the direct interaction between cells and the surface of candidate metals, which will occur following the implantation of BRS within tissue [76]. To promote cell adhesion to candidate metals, cells are typically dispensed onto metal samples and cultured for 24–72 h, similar to the approach used in extract tests [26,45,65,66]. Whilst this is common practice, refinements to this procedure have been developed to better control cell adhesion. For example, Mao et al. [69] designed an artificial scaffold in which cells were cultured within, to more closely replicate the complex structure of the arterial vasculature.

Similar to extract tests, direct contact tests are primarily performed to evaluate the cytotoxicity of metallic BRS [45,65,66]. This is accomplished by observing cell morphology, migration and lysis following culture on candidate metals through either electron microscopy or immunofluorescent staining in conjunction with fluorescent microscopy, as detailed in Table 5 [45,65,66,69]. Direct contact tests are further used to examine the interactions between candidate metals and blood samples, providing insight into their haemocompatibility in accordance with ISO 10993-4 guidelines [23,45,72,74]. For the evaluation of haemocompatibility, two main experimental assays are performed: haemolysis assays and platelet adhesion/thrombus formation assays [23,45,72,74]. To examine the haemolytic effects of candidate metals, blood samples are incubated in direct contact with the candidate metals for a period of 1–2 h, after which the extent of haemolysis is quantified via spectrophotometry [23,45,72,74]. Similarly, to assess the thrombotic effects of candidate metals, platelet-rich-plasma is applied directly onto candidate metals for a similar duration to extract tests (approximately 24 h), before examining for the adhesion and morphology of adhered platelets utilising scanning electron microscopy [23,45,72,74].

**Table 5:** **Common experiments to evaluate the biocompatibility of bioresorbable metals using direct contact tests**.

Category of biocompatibility	ISO standard	Biological Response	Relevant Tests	Refs
Cytotoxicity	ISO 10993-5	Morphological changes Cell Lysis	Scanning Electron Microscopy Fluorescence Microscopy	[45,[86], [87], [88]]
Haemocompatibility	ISO 10993-4	Haemolysis	Haemolysis assays	[44,45,70,74]
		Thrombosis	Platelet adhesion assay	

Direct contact methods are thus a valuable complimentary model to extract tests, as they enable researchers to observe the direct interactions cells have with candidate metals, a feature which extract tests fail to replicate. However, it should be noted that candidate metals used in direct contact tests are typically thicker and structurally simpler than stents used in a clinical setting, thus limiting their ability to accurately mimic physiological conditions [66,67]. Moreover, direct contact tests typically involve the culture of a singular cell type on candidate metals, which does not reflect the complex multicellular environment of tissues [66,67]. These observations demonstrate that there is thus a need for further optimisation of direct contact tests to more accurately replicate the physiological conditions of the implantation of BRS [[64], [65], [66],^69^].

### 2.3 Role of indirect contact tests in evaluating biocompatibility

Indirect contact tests are a less-commonly utilised in vitro model in which candidate metals are placed on top of a barrier which separates the material from a cell monolayer [26]. This barrier may be compromised of a thin layer of agar lying directly on top of cells, or as a filter within a cell-culture insert placed above cells (Fig. 2B–iii) [89,90]. The presence of this barrier decreases the concentration of degradation products that reaches the cellular monolayers, thus lowering the concentration of extracts cells are exposed to during culture without the need for manual dilution. Typically, indirect contact tests are utilised to evaluate the toxicity of medical implants which contain materials of a known cytotoxicity, or materials that will not directly come into contact with tissue, such as dental implants [89,90]. Whilst useful to evaluate the biocompatibility of these other medical implants, indirect contact tests do not adequately model the implantation of a BRS directly onto the arterial wall, which involves a higher degree of direct cell contact. As such, indirect contact tests have not been used in current literature to evaluate the biocompatibility of metallic BRS. However, indirect contact models may be valuable for investigating the effects of degradation products released by candidate metals on downstream blood vessels or organ systems that do not come into direct contact with the implant, thereby providing insight into the systemic effects of metallic BRS.

### 2.4 Considerations and limitations for in vitro models

As a preclinical model, in vitro cellular models offer a low-cost, time-efficient and simplistic experimental model to evaluate the biocompatibility of metallic BRS. In turn, the importance of in vitro testing cannot be understated. However, there are notable gaps and areas for improvement in the use of in vitro models concerning current research efforts on metallic BRS. One key area of concern amongst previous literature is a notable heterogeneity between employed cell lines. Whilst international standards such as ISO 10993-5 provide recommended cell lines for use in in vitro models, they allow researchers a degree of autonomy in their choice of cellular models and do not specify where these cell lines must originate [21]. As a result, there has been a wide range of cells previously utilised to assess the biocompatibility of bioresorbable metals, examples of which are outlined in Table 6, which vary in both their type (i.e. endothelial cells, smooth muscle cells or fibroblasts) and origin (i.e. human or murine) [37,44,59,73,75].

**Table 6:** Commonly utilised cell lines used in in vitro models for evaluating the biocompatibility of metallic BRS.

Cell Line	Cell Type	Origin	Refs
Human Coronary Artery Endothelial Cells (HCAEC)	Primary	Human, artery	[44,65,67]
Human Coronary Artery Smooth Muscle Cells (HCASMC)	Primary	Human, artery	[65,70]
Human Dermal Fibroblasts (HDF)	Primary	Human, epidermis	[65]
L929 Fibroblasts	Immortalised cell line	Mouse, areolar/adipose tissue	[59,62,66,68,72]
Human Umbilical Vein Endothelial Cells (HUVEC)	Primary	Human, umbilical cord	[69,75]
U937 Monocyte	Immortalised cell line	Human, lymphoma	[37]
Ea.hy926 Endothelial cells	Immortalised cell line	Human, umbilical cord	[73]

The specific reasons for selecting certain cell lines are not always explicitly stated within the literature, but they are likely influenced by factors such as cost, availability and the preference for primary or immortalised cells. Whilst some variability between the cell lines utilised between studies is expected, it has been previously documented that the cellular tolerance to metallic ions can vary between cell lines and types due to metabolic differences within cells [79,91]. Additionally, some of the previously utilised cells, such as L929 fibroblast cell lines, which originate from mouse adipose tissue, do not originate from the vascular system of a human [59,62,66,68,72]. Consequently, it is challenging to make comparisons between studies that have utilised varying cell types and originate from different organs, as it is unclear whether the reported effects of candidate metals are due to the metal itself or the inherent tolerance of the chosen cell line to its degradation products. As such, to accurately model the physiological response of the tissue, it is recommended to use cells derived from the human cardiovascular system (e.g. arterial endothelial cells, vascular smooth muscle cells, fibroblasts) as these more closely resemble the cellular environment of the implanted stents [44,65,67,92,93]. Moreover, the incorporation of robust internal controls within experimental assays alongside rigorous statistical analysis. Moreover, ensuring the proper use of robust internal controls (i.e. appropriate blank, positive, negative and experimental groups), combined with rigorous statistical analysis, is essential to ensure the validity of findings, regardless of the heterogeneity between cell lines [94]. To ensure this, it is recommended that investigators consistently refer to standardised experimental frameworks including ISO 10993-12 and the ARRIVE frameworks [94,95].

Another notable gap within current in vitro models is an imbalance between the aspects of biocompatibility investigated in vitro. Whilst the immunogenicity and haemocompatibility of candidate metallic BRS have been briefly explored in vitro, predominantly, in vitro studies tend to focus their research efforts on evaluating the cytotoxicity of candidate metals [44,45,58,59,62,[65], [66], [67], [68], [69], [70], [71], [72], [73], [74], [75]]. Moreover, whilst standards such as ISO 10993-3 (2022) provide recommendations on evaluating the genotoxicity and carcinogenicity of biomedical implants, there is a current lack of studies that have investigated these aspects of biocompatibility concerning metallic BRS in vitro [22]. Whilst these aspects of biocompatibility have been demonstrated using in vivo models, it is unclear why there is a lack of in vitro testing of the carcinogenicity and genotoxicity of metallic BRS [[96], [97], [98]]. One possible explanation may be due to the strong emphasis placed on demonstrating the cytocompatibility of metallic BRS when seeking regulatory approval, as well as the standardised protocols and relative ease of performing cytotoxicity assays [21,26].

To comprehensively understand the biocompatibility of metallic BRS, it is recommended to investigate, even if briefly, each key aspect of biocompatibility in vitro. This will aid researchers in building a stronger foundational understanding of the properties of candidate metals and ensure that any unexpected adverse effects of materials are identified before progressing to more complex preclinical models. ISO 10993-3 clarifies that genotoxicity and carcinogenicity testing is only necessary when candidate materials are known to exhibit genotoxic or carcinogenic effects, or when insufficient prior data on the candidate material exists [22]. Whilst the foundational research into metallic BRS is extensive, it has been shown that excess metallic ions such as copper, aluminium, or iron, can induce unexpected carcinogenicity and genotoxic effects, including genetic damage [22,96,97,99]. Cost-effective assays with existing standardised protocols for investigating the genotoxicity and carcinogenicity of metallic BRS in vitro, and are supported by ISO 10993-3, include the micronucleus, comet or γ-H2AX assay which detect signs of 10.13039/100026054DNA damage and breakage [22,[100], [101], [102]].

In summary, preclinical in vitro cellular models offer a low-cost, time-efficient and simplistic experimental model to evaluate the biocompatibility of metallic BRS, and the importance of the use of these models cannot be understated. When considering which type of in vitro test to perform, investigators are recommended to choose a model appropriate to the aspects of biocompatibility to be investigated (i.e. cell-material or cell-degradation product interactions). For a comprehensive understanding of the biocompatibility of metallic BRS, it is advisable to investigate each key aspect in vitro using a combination of both direct and extract tests. This will aid researchers in building a stronger foundational understanding of the properties of candidate metals and ensure that any unexpected adverse effects are identified before moving on to more complex preclinical models.

## 3 In vivo animal models to evaluate the biocompatibility of metallic bioresorbable stents

**Fig. 3:**
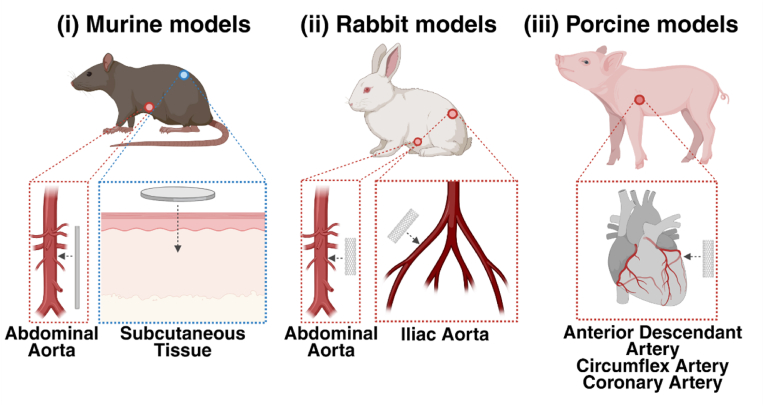
Animal models utilised to evaluate the biocompatibility of metallic BRS in vivo. A combination of small and large animal models has been utilised to assess the biocompatibility of candidate metallic BRS. Small models, such as (i) rodent (i.e. rats or mice): and (ii) rabbit, have been used to initially assess the biocompatibility of metallic BRS. In these smaller models, candidate metals have been implanted in the abdominal aorta, subcutaneous tissue or iliac artery in the form of either samples of the metal (present as a disc or wire), or as a whole stent comprised of the candidate metal. (iii) Large animal models, such as pigs, have been utilised to assess the performance and biocompatibility of stents over prolonged periods. Due to the larger vasculature and organs, pig models allow stents to be implanted in locations similar to those in humans (i.e. the coronary arteries).

The use of animal models in evaluating the safety and efficacy of medical implants, including cardiovascular stents, is a well-established practice utilised for over 40 years [103]. In the field of cardiology, a plethora of animal species, including rodents, porcine, ovine, canine and non-human primates, have been previously used as models to investigate the pathophysiology of cardiovascular disease and potential therapeutics, which have been previously summarised by Camacho et al. and Perkins et al. [104,105]. In terms of evaluating the biocompatibility of metallic BRS, animal models are utilised specifically to understand the vascular responses to the candidate metal, as well as the systemic toxic effects and complications these materials impose whilst degrading [60,81,[106], [107], [108], [109], [110], [111]]. As such, direct contact/implantation animal models have been most commonly utilised, which involve the implantation of candidate metallic BRS within the subcutaneous tissue and the aorta of murine, rabbit and porcine animals to evaluate their biocompatibility (Fig. 3) [39,110,112,113].

These animal models provide a key benefit to researchers as they enable the observation of the biological interactions these materials have within the complex and dynamic multicellular environment of tissue in vivo [104,114]. Moreover, the effects of breakdown products released from candidate metals as they migrate throughout the bloodstream and reach downstream organs can be observed through the use of these animal models, providing insight into the systemic toxicity of candidate metals [39,42,60,104,114]. Animal models are thus invaluable in providing insight into the potential long-term success of a candidate metallic BRS following their implantation in vivo, and have been used in the evaluation of BRS comprised of magnesium, zinc, iron and molybdenum alloys [60,81,[106], [107], [108], [109], [110], [111]]. Rather than comprehensively evaluating all aspects of biocompatibility, previous investigations have predominantly focused on evaluating the immunogenicity and carcinogenicity of implants through the post-mortem analyses of immune cell infiltration, tissue necrosis and/or fibrosis [24,115].

Complementing in vitro models, internationally recognised guidelines, specifically the ARRIVE guidelines and ISO articles 10993-2 and 10993-6, have been established to provide recommendations for the experimental design and conduct of in vivo biocompatibility studies [22,95,115]. They outline key aspects such as study duration, sample sizes and the appropriate methodologies for post-mortem examination of both animals and implanted materials [24,115]. However, it should be noted that whilst these standards provide experimental frameworks and guidance for implantations within the subcutaneous tissue, brain, muscle and bone, they do not provide any recommendations specific for evaluating the biocompatibility of materials within the vasculature [24,115]. Further, the ISO series 10993 does not comprehensively provide guidance on designing in vivo studies to evaluate the biocompatibility of metallic BRS. Consequently, there is a large variability between the experimental design of in vivo studies in the current literature, including the choice of animal model, the structure of the implanted candidate metal and the post-mortem tests performed to evaluate biocompatibility [81,[108], [109], [110]].

### 3.1 Evaluating short-term biocompatibility of BRS with murine models

Due to their relatively low cost, housing requirements and ease of handling, rodent models (i.e. rats or mice) have been extensively utilised to evaluate the local effects of implanted candidate metallic BRS in vivo [114]. Previously, candidate bioresorbable metals have been implanted either in the abdominal aorta to study their biological interactions within the cardiovascular environment, or in subcutaneous tissue to examine the systemic effects of metal degradation on distal organs such as the brain, liver and kidneys (Fig. 3 i.) [60,64,83,106,107,116]. Alternatively, instead of implantation, alloy extract solutions containing the degradation products released from candidate metallic BRS have been directly injected into animals to observe effects of the degradation products themselves, as demonstrated by Wang et al. [117] (Fig. 3 iii).

**Fig. 4:**
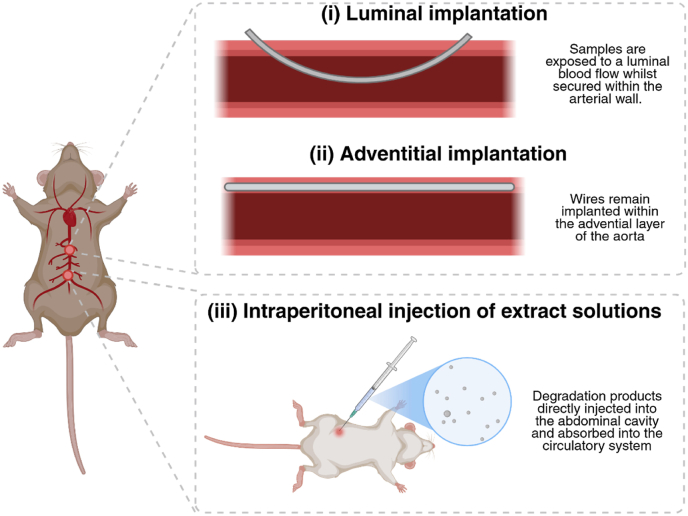
Methods of implantation of bioresorbable metal samples within the aorta of murine animal models. Samples of candidate metals (typically as wires or rods) can be either implanted (i) within the aortic lumen whilst secured into the aortic wall or (ii) within the adventitial layer of the aortic wall. Alternatively, (iii) solutions containing degradation products released from candidate metals can be intraperitoneally injected to examine their systemic effects. The luminal implantation models enable investigators to examine interactions between candidate materials and blood components while simultaneously assessing metal degradation under continuous blood flow. Adventitial implantation models provide insight into the interactions between candidate metals and the tissue present within the medial and adventitial layers of the aortic wall.

For arterial implantation, samples of candidate metals are typically extruded into thin wires, approximately 10–15 mm in length and 0.25–0.5 mm in diameter, simulating a singular strut of a stent [39,42,60]. Once prepared, wire samples are manipulated into the lumen of the caudal descending abdominal aorta and vena cava (diameter of approximately 0.8–0.9 mm) to immerse samples in flowing blood (Fig. 4 i), simulating the initial environment a stent will encounter [60,106,107,116,118]. Alternatively, the wires may be implanted directly within the adventitial layer of the arterial wall (Fig. 4, ii) to expose the stents to arterial extracellular matrix and cells to simulate the long-term environment a stent will be exposed to Ref. [60]. Attempts have also been made to implant whole stents (approximately 2.5-3 x 0.8–1 mm with a strut thickness of 0.06 mm) comprised of candidate metals, such as by Douglas et al. [119] and Chamberlain et al. [120] within the thoracic aorta of mice (approximately 1 mm in diameter) utilising traditionally coronary balloon angiography, to examine the efficacy of a candidate metallic BRS within a design which more closely emulates the clinical design of BRS [110,121]. For implantation within the subcutaneous tissue, samples have previously been drawn into wires as detailed above, or into small disc samples (approximately 6.5 mm in diameter and 2 mm in thickness) before being implanted within the scapular or lumbar region of the animal [81,83] (see Fig. 4).

After implantation, animals are left to recover and monitored for 2–12 months; after which tissue and bodily fluid (including blood, urine and serum) are collected for histological and biochemical analysis [83,107]. An advantage of in vivo models is that the degradation of the implanted candidate metal can be monitored over time using non-invasive imaging techniques, such as optical coherence tomography (OCT), as well as tracking the concentration of metal ions in the blood through fluorometric assays or hematologic analysers [83,107,110]. Together, these pre- and post-mortem analyses provide insight into the health and immune response across various organ systems, and enable assessment of the potential for candidate metals to induce complications following implantation [60,106,107,116].

### 3.2 Rabbit models provide an alternative to murine models

Whilst murine models are cost-effective and have less demanding housing requirements, their arterial vasculature is significantly smaller than that of a human, as detailed within Table 7 [[122], [123], [124]]. In comparison, rabbit models are an alternative small animal model which remains cost-effective and widely available, whilst also possessing vasculature more similar to that of humans (approximately 2.8mm–3.7 mm in diameter) [56,125]. With this advantage, rabbit models present a promising small-animal model for evaluating the biocompatibility of metallic BRS in vivo.

**Table 7:** Comparison of the arterial vasculature between animal models and humans.

Organism	Arterial Diameter	Ref.
Mice	Abdominal aorta; 1–2 mm	[122,123]
Rat	Abdominal aorta; 1.3–1.5 mm	[126,127]
Rabbit	Iliac artery; 2.1–3.7 mm	[125]
Pig	Left coronary artery; 2.7–6.8 mm	[128,129]
	Ascending aorta; 20–24 mm	
	Pulmonary artery; 23–25 mm	
Human	Right coronary artery; 2.8–4.2 mm Left main artery; 4.5–5 mm	[124]

For implantation within the vasculature of a rabbit, candidate metals are typically prepared into whole stents (approximately 8–15 mm long, 50 μm thick) to more accurately simulate the clinical design of candidate stents [108,112]. Once prepared, these stents are implanted utilising traditional balloon angioplasty into the iliac or abdominal aorta of rabbits, arterial locations which more closely emulates the clinical implantation of a stent itself of the human aorta (Fig. 3, ii) [108,112,130]. Following implantation, rabbits are closely monitored for approximately 3–24 months before collecting and examining aortic tissue through histological and immunohistochemical analysis to examine for signs of chronic inflammatory responses or morphological changes, similar to rodent implantation models [108,112,130]. Overall, rabbit models provide a promising option for in vivo studies to assess the biocompatibility of metallic BRS, however, their use is limited by the need for ample housing space and growing societal pressure to reduce their use for scientific purposes [56,114].

### 3.3 Evaluating long-term biocompatibility with porcine models

Porcine models are a highly favourable animal model when evaluating the biocompatibility of metallic BRS due to the anatomical similarities between porcine and human vasculature, both in arterial diameter size, diameter and length [131,132]. This larger vasculature enables stents of various sizes and designs (typically 150–180 μM stent strut thickness, 15–20 mm in total scaffold length) to be implanted and consequently evaluated in vivo [56,82,84,121]. Compared to the use of disc, wire or smaller stent samples which are implanted within smaller animal models, these BRS that are implanted within pig models reflect the clinical design of a stent to enable for any unexpected complications with these prototype stent designs to be observed [56,82,84,121].This results in the reported biocompatibility of candidate metals more closely reflecting the physiological conditions of the human vasculature, as well as allowing for the design of the stent to be prototyped before clinical trials [56,82,84,121]. As such, due to the higher cost and housing requirements of a large animal, rather than evaluating early-stage toxicity, porcine models are typically utilised to evaluate late-stage complications associated with candidate metallic BRS, such as re-endothelisation, restenosis or stent-induced thrombosis [56,82,84,121].

To comprehensively understand the biocompatibility of candidate metallic BRS within the coronary vasculature, BRS comprised of candidate metals are typically deployed in the left anterior descendant artery, the left circumflex artery and the right coronary artery to capture responses across the major coronary branches (Fig. 3, iii) [82,83,87,99]. These candidate stents are deployed into arteries typically through the use of traditional coronary balloon angioplasty similar to procedures performed clinically [84,121,133]. Following implantation, stents remain typically implanted for 6–24 months, with the placement and degradation of the stent monitored through the use of intravascular ultrasounds (IVUS) or OCT [84,109,110,121]. As with other animal models, at the conclusion of the study period, aortic tissue surrounding the implanted stent undergoes histological and immunohistochemical analysis to examine for indications of significant inflammation, changes in vessel morphometry or thrombus formation [84,110,121,133]. In short, porcine models are a highly relied upon animal model for evaluating the biocompatibility of candidate metallic BRS in vivo due to their similar cardiovascular physiology to humans [56,114]. However, a higher economic cost, larger demand for housing space and a growing societal pressure to reduce the use of porcine models hinders their widescale use, limiting the number of studies that employ them [114].

### 3.4 Post-mortem analysis of in vivo models for the evaluation of biocompatibility

Regardless of animal model used, post-mortem analysis of the implantation site, along with surrounding organ systems, is paramount for evaluating the effects the candidate bioresorbable metal has on the animal. This analysis typically involves examining collected tissues and systemic fluids (e.g. blood or serum) for any signs of inflammation, tumour formation or thrombosis [64,86,111,119,120]. To accomplish this, a combination of histological and immunohistochemical staining is used, as detailed in Table 8. Histomorphometry is the most commonly employed technique for observing the general morphology of tissue, including specific tissue elements such as immune cell recruitment or fibrous tissue formation, which indicate the overall extent of inflammation [81,[108], [109], [110]]. As such, haematoxylin & eosin (H&E) staining is utilised extensively tissue samples both surrounding the implant and within downstream organs to examine changes in tissue morphology [81,[108], [109], [110]]. Other commonly used stains, including Verhoeff-Van Gieson (VVG), Giemsa and Toluidine blue, have also been utilised to visualise elastic stretching of tissue, differentiated blood cells and mast cell granulation, respectively [84,107,133]. To further characterise specific cell types within tissues, immunohistochemistry targeting cellular markers such as CD31, alpha-smooth actin, CD68, CD206 and CD11b has been used. This approach enables the identification of endothelial cells, muscle fibres, monocytes or other cell populations, as well as quantification through cell counting [109,119,133]. In addition to staining, tissue samples have been analysed utilising OCT and SEM to provide high resolution, 3D and label free imaging that offers complementary insights into cellular interactions and tissue architecture with enhanced detail beyond conventional histology [39,82,112]. Together, the examination of the tissues overall morphology, along with the identification of cells within tissue, helps determine the overall status of the organ system and indicates whether any significant immunological responses have occurred as a result of implantation of the candidate metal.

**Table 8:** In vivo analysis techniques used to evaluate the biocompatibility of metallic BRS in accordance with ISO 10993-6.

Category of biocompatibility	Analysis Technique	Rationale of use	Refs
Immunogenicity	Histological stains; H&E, VVG, Toluidine Blue, VWF, Giemsa.	Identification of structural elements within tissue samples, as well as the overall extent of inflammation.	[42,60,[81], [82], [83], [84],^86^,^106^,^107^,[109], [110], [111], [112],^116^,^133^,^134^]
	Immunohistochemical markers; CD31, CD68, CD206, CD11,	Identification of different cell types within a tissue.	[81,108,116]
	α-SMA		
	Scanning electron microscopy; optical coherence tomography	High-resolution imaging and morphometric analysis of tissue architecture and implant-tissue interactions.	[39,42,82,108,110,112,133]
	Full blood counts	Quantification of white and red blood cell counts to evaluate immune responses or anaemia following stent implantation	[83]
Systemic Toxicity	Protein Quantification; quantification of haemoglobin, aminotransferase, alkaline phosphate, alanine transaminase in blood	Observation of immune responses and toxicity following implantation	[107]

### 3.5 Considerations and recommended modifications for in vivo preclinical models

In summary, animal models represent indispensable preclinical research platforms that enables investigators to comprehensively assess the long-term degradation, safety and efficacy of candidate metallic BRS. Within the experimental design phase of in vivo studies, it is pertinent that researchers consider various characteristics when deciding upon which animal to utilise (Table 9)**.** Of these factors, investigators must review the applicability of the animal model in relation to the predicted degradation rate of the candidate metal and the overall duration of the study. A key mechanism of the degradation of metallic BRS in vivo is the evolution of hydrogen gas and absorption of oxygen by metallic ions within the candidate metal, leading to their detachment from the implanted sample and release into the bloodstream [19,56]. The varying blood-oxygen content and metabolic rates across different animal species have been shown to influence metal degradation, with smaller animals typically exhibiting higher degradation rates than larger animals [135,136]. It is therefore highly recommended that investigators select an animal model that accurately reflects the degradation of metallic BRS within the human vasculature, adjusting their choice based on the intended study duration. Larger animals are essential for long-term studies, while smaller animals are suitable for shorter-term studies [135,136]. Additionally, the choice of model may vary depending on the stage of preclinical evaluation, with smaller animals typically used for early biocompatibility screening and larger models reserved for later-stage efficacy and performance testing [114,137].

**Table 9:** Comparative characteristics of small and large animal models relative to humans.

Organism	Cost	Housing Requirements	Metabolic Rates	Physiological and anatomical relevance	Ref
Mice	Cheap, readily available	Easy handling, breeding and less demanding care	Significantly higher than human	Significantly smaller anatomical size, low physiological similarity to humans	[138,139]
Rat	Cheap, readily available	Easy handling, breeding and less demanding care	Significantly higher than human	Significantly smaller anatomical size, low physiological similarity to humans	[104,139]
Rabbit	Affordable, readily available	Easy handling, breeding, moderate space demand	Higher than human	Poor similarity to the human and muscular arteries than a human; larger anatomical size, low physiological similarity to humans	[104,139]
Pig	High cost	Large space demand, complicated handling	Similar to human, lower oxygen affinity	Similar physiology to humans, faster growth rates and high body weight	[[139], [140], [141]]

In addition to the size of the animal itself, the genetic strain of the animal model should also be considered. Whilst various genetically modified strains, such as atherosclerosis-prone, apolipoprotein-E (ApoE) knockouts are available for use, current literature typically use wild-type of genetically healthy species of animals [120,130,132,142]. Given that cardiovascular stents are typically implanted in patients with severe CVD, the morphology of the arterial environment in these individuals would differ significantly from that of healthy animals [143]. Consequently, the biocompatibility of candidate bioresorbable metals within atherosclerotic arteries remains largely unexamined, highlighting a crucial gap in current in vivo models and a potential for improvement in the evaluation of BRS materials.

## 4 Future direction for preclinical research models and clinical trials for metallic BRS

### 4.1 Three-dimensional cellular models have the potential to enhance in vitro research

In current literature, the use of two-dimensional (2D) cellular monolayers used to evaluate the biocompatibility of metallic BRS in vitro has limitations in replicating the complex three-dimensional (3D) structure and multicellular interactions within tissue [144]. This often results to an over-reliance into the use of animal models to validate the observations made in vitro [137,144]. However, the high economic burden that these animal models impose, as well as changing societal opinions regarding the ethical use of animal models has led to a growing demand for alternative preclinical research models [137,144].

**Fig. 5:**
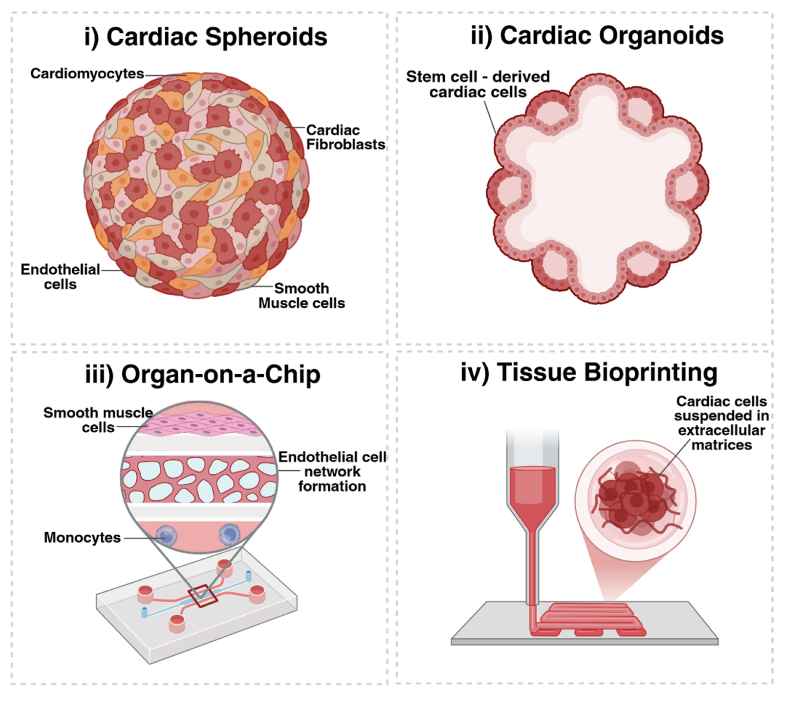
3D Cardiovascular Cell Culture Models. (i) Cardiac spheroids consist of cardiac cells including fibroblasts, cardiomyocytes, endothelial or smooth muscle cells, grown into spherical. (ii) Cardiac organoids are similar to spheroids but instead rely on the use of stem cells to form self-assembling aggregates of cells. (iii) Organ-on-a-chip technology (consists of cardiac cells grown within artificial microfluidic structures that expose cells to a flow of liquid. (iv) 3D bioprinting involves the construction of artificial 3D printed tissues incorporating the use of cardiovascular cells and bioinks.

A promising alternative to current in vitro models which have begun to be utilised in drug development and testing are 3D cell culture models. These advanced cell culture models are developed in environments that promote 3D growth, either through the use of supporting scaffolds or by altering traditional culture conditions [145]. A variety of 3D cellular models utilising cardiovascular cells have been established and are increasingly used to model CVD and aid drug discovery and development for potential therapeutics [[145], [146], [147]]. These include cardiac spheroids (Fig. 5, i), which are comprised of a combination of cardiovascular endothelial cells, fibroblasts, smooth muscle cells or cardiomyocytes cultured in a spherical form that replicates the human heart microenvironment at the cellular level [144,[148], [149], [150], [151]]. Alternatively, cardiac organoids, comprised of self-organised structures of stem cells that have differentiated into various cardiac cells have been used to replicate the characteristics of the heart (Fig. 5, ii), [144,149]. Artificial culture vessels, such as microfluidic platforms, have also been utilised to create 3D ‘heart-on-a-chip’ or ‘vessel-on-a-chip’ cellular models (Fig. 5, iii) [144,148]. These models consist of cells cultured within an extracellular matrix whilst being exposed to a flow of cell culture medium that replicates blood flow through the vasculature in vivo [144,148]. Emerging techniques such as tissue bioprinting and cardiac tissue engineering (Fig. 5, iv) have also been gaining traction. These methods involve the use of 3D printing using cardiac cells and bio-inks to construct the complex architecture and function of the cardiovascular system [148,151].

3D cellular models have increasingly been integrated into experimental assays to investigate biocompatibility. For example, cardiac organoids have been used in conjunction with fluorescence microscopy to evaluate the toxicity of doxorubicin (DOX), a known cardiotoxin. [149,152,153]. It is thus unsurprising that 3D cell models have begun to be incorporated into biomaterials research, such as demonstrated by Dhall et al., who developed a 3D dental implant-on-a-chip model to examine the interactions between a dental implant and surrounding soft tissues [154]. 3D cellular models have yet to be utilised in studies evaluating the biocompatibility of metallic BRS, presenting significant potential for their incorporation into preclinical assessments. However, before adopting these complex models, investigators should compare the benefits of 3D cellular models with currently available 2D monoculture assays (which has been previously reviewed by Kapałczyńska et al. [155], and herein summarised within Table 10), to determine whether 3D cellular models are necessary for their investigations. Whilst 3D cellular models more closely mimic the complex morphology of the human vasculature, they are typically associated with a higher cost, longer growth periods and more complex, less-standardised experimental protocols [145,150]. In comparison, 2D cellular monocultures are significantly cheaper and have standardised experimental protocols, which lead to a higher reproducibility of results across experiments [155]. Moreover, recent policy updates from the United States National Institutes of Health (NIH) announced in July 2025, which now requires that research proposals include consideration of in vitro methodologies complement in vivo animal models, highlights the potential of 3D cellular models as a promising future direction for in vitro research on metallic BRS; however, their applicability must be carefully considered before incorporation into studies.

**Table 10:** Comparison of 2D and 3D cellular models.

Type of Culture	Advantages	Disadvantages	Ref
2D	Cheap, readily available, standardised protocols, shorter culture time, higher reproducibility and more simplistic findings	No presence of cell-cell interactions or extracellular matrix environments, does not mimic the physiology of tissue	[[155], [156], [157], [158], [159]]
3D	Possess cell-cell and cell-extracellular matrix interactions, more closely mimic the physiology of human tissue.	Longer culture time, lower reproducibility, complicated experimental protocols, and harder to interpret findings	

### 4.2 Evaluating the performance of metallic BRS within a clinical environment

Evaluating the therapeutic effectivity of candidate metallic BRS in a clinical setting is essential to identify any unexpected complications following implantation and to validate preclinical findings [55]. Few metallic BRS have proceeded to clinical trials, with the most extensively studied being the magnesium-based BRS produced by Biotronik. This device has been evaluated across multiple clinical trials, including PROGRESS-AMS and BIOSOLVE-I-IV programs, which have assessed its safety, efficacy and long-term performance in patients [[48], [49], [50],^55^,[160], [161], [162]]. These large-cohort clinical trials have demonstrated that these stents perform comparably to currently utilised DES, with less <5 % of patients experiencing complications associated with these stents such as thrombosis or restenosis, with trials still ongoing [[48], [49], [50],^55^,[160], [161], [162]]. Whilst these initial findings were promising, rare occurrences of early scaffold breakdown have occurred following the implantation of these stents, resulting in the design of these stents continuing to be refined [163,164].

Unlike preclinical studies, the experimental design of these clinical focuses on identifying clinical complications associated with the BRS rather than specific aspects of biocompatibility [[48], [49], [50],[160], [161], [162]]. Within these trials, patients with CVD including ischemic heart disease, myocardial infarction or critical limb ischemia, are selected to undergo percutaneous coronary intervention (PCI) and BRS implantation [[48], [49], [50],[160], [161], [162]]. Following PCI, the vasculature of the patients (such as the vessel lumen and diameter), as well as the status of the stent, are characterised using IVUS or intra-arterial digital subtraction angiography (DSA), which involves the use of a contrast dye and x-ray images to observe blood vessels [48,160,162]. Clinical assessments of patients are performed periodically for 1–36 months following BRS implantation which involve a combination of qualitative and quantitative tests to examine the status of the patient and the stent itself [[48], [49], [50],[160], [161], [162]]. Qualitative assessments of patient condition involved the use of questionnaires to assess if patients required medication, further hospitalisation or experienced any adverse cardiac events following stent implantation [[48], [49], [50]]. The condition of each patient's vasculature was then examined using coronary angiographies, IVUS or OCT to determine any changes in arterial status or any changes in the condition of the stent itself [[48], [49], [50],^160^,^162^]. Certain studies, such as Bosiers et al. (2009) opted to perform specialised techniques to analyse arterial status, such as utilising colour flow duplex ultrasounds (CFDU) to investigate implanted stents [48]. Similarly, Sabate et al. (2019) performed intracoronary infusions of acetylcholine to analyse cell status following BRS implantation [50]. After the trial period, the condition of each participant in the study cohorts, as well as the outcome of each implanted BRS was collected into a registry to assess the overall outcome and performance of the candidate metallic BRS [[48], [49], [50],^160^,^162^]. Clinical trials are therefore necessary to capture complex patient responses and long-term outcomes that preclinical models cannot fully predict, ensuring candidate metallic BRS are both safe and effective in diverse patient populations.

## 5 Conclusion and future perspectives

Metallic bioresorbable stents present a promising, innovative solution to the current complications associated with prevailing corrosion-resistant and drug-eluting stents. An overview of the current progress of the leading metallic BRS including magnesium, iron and zinc-based alloys demonstrated the need to further optimise and refine the design of BRS to further improve their clinical performance. Given their promising potential, it is crucial to thoroughly investigate the biocompatibility of the BRS to ensure their safety and effectiveness. This review presents the preclinical models currently used to evaluate these materials, highlighting their strengths and limitations to guide future research.

Preclinical studies provide valuable insight into the interactions between candidate metals and the arterial environment which assist in the optimisation of the design of these BRS. However, a lack of cohesion in the experimental design of preclinical studies, as well as generalised international standards, creates challenges in building a comprehensive understanding of the biocompatibility of these candidate metals. In vitro preclinical models, including extract and direct-contact cellular models, provide a time-efficient and cost-effective method of evaluating the initial toxicity of candidate metals. Although these methods provide useful preliminary data, many rely on animal-derived cells and simplified 2D cellular monolayers which may not fully replicate human tissue physiology. To enhance translational relevance, researchers should prioritise validation with human-derived cells and consider 3D culture systems to better mimic in vivo conditions.

This review further provided a comparison of current animal models utilised to evaluate the biocompatibility of metallic BRS in vivo. Whilst current in vivo models provide valuable insight into the biological interactions that candidate metals have with biological tissue and distal organs, researchers need to carefully select the appropriate animal model depending on the stage and goal of their study. For example, rodent models offer early, cost-effective insights into biocompatibility, whereas larger animal models are better suited for later-stage evaluation of device performance and clinical relevance. Moreover, it is beneficial to explore if the reported biocompatibility of candidate metals will differ within a diseased animal model, as pathological conditions can significantly influence, the biological response to implanted materials.

In summary, BRS hold great promise as next-generation cardiovascular implants, and this review highlights that combining 2D and 3D in vitro models with animal in vivo models is recommended to develop a comprehensive understanding of the biological interactions between metals and the arterial environment, supporting rigorous biocompatibility evaluation needed before usage in clinical settings.

## CRediT authorship contribution statement

**Samuel Hansen:** Writing – review & editing, Writing – original draft. **Thuy Anh Bui:** Writing – review & editing. **Xiaoxue Xu:** Writing – review & editing, Supervision, Conceptualization. **Kristine McGrath:** Writing – review & editing, Supervision, Conceptualization.

## Ethics approval and consent to participate

Not applicable for this review.

## Declaration of competing interest

The authors declare the following financial interests/personal relationships which may be considered as potential competing interests: Xiaoxue Xu is an associate editor for Bioactive Materials and was not involved in the editorial review or the decision to publish this article. All authors declare that there are no other competing interests.
